# Rapidly Progressive Dementia After Platelet-Rich Plasma Therapy: A Case of Sporadic Creutzfeldt-Jakob Disease in the Context of Chronic Ehrlichiosis

**DOI:** 10.7759/cureus.91152

**Published:** 2025-08-27

**Authors:** Mili Dave, Peter Le

**Affiliations:** 1 Internal Medicine, University of North Carolina School of Medicine, University of North Carolina at Chapel Hill, Chapel Hill, USA; 2 Hospital Medicine, UNC Health REX Hospital, Raleigh, USA

**Keywords:** ehrlichiosis, platelet-rich plasma (prp), prion diseases, rapidly progressive dementia, scjd

## Abstract

Prion diseases are fatal neurological disorders characterized by the accumulation of abnormal proteins in the central nervous system, leading to rapid neurodegeneration. We present a male his 70s who developed rapidly progressive dementia three weeks after a routine platelet-rich plasma (PRP) injection for chronic hip pain. MRI revealed multifocal cortical diffusion restriction involving the basal ganglia. CSF analysis via real-time quaking-induced conversion (RT-QuIC) confirmed prion disease, with elevated T-tau and 14-3-3 gamma proteins. He also tested seropositive for Ehrlichia, though other infectious causes were ruled out. Despite treatment with IV immunoglobulins (IGs) and antibiotics, his condition declined with worsening myoclonus and hyperreflexia. He died one week after discharge to hospice, six weeks after symptom onset. This case demonstrates several classic clinical features of sporadic Creutzfeldt-Jacob disease (sCJD) and raises the possibility that an acute inflammatory response from PRP injection may have triggered sCJD in the setting of a pre-existing chronic inflammatory state from tick-borne infection.

## Introduction

Creutzfeldt-Jacob disease (CJD) is a rare and fatal neurodegenerative disease resulting from the accumulation of misfolded prion proteins (PrP^Sc^) in the central nervous system. These conformationally altered proteins lead to prion aggregation, widespread neuronal loss, neurotoxic spongiform degeneration and gliosis [1]. CJD is typically classified into sporadic, genetic, and acquired forms, with the sporadic subtype representing about 85% of all cases [2]. Most often, patients present in the sixth to eight decades of life with rapidly progressive dementia and multi-axial neuronal losses including myoclonus, cerebellar, and extrapyramidal signs; visual disturbances; and cognitive/functional impairment [3]. In advanced stages of the disease, patients often exhibit akinetic mutism and severe diffuse rigidity with eventual death usually within one year of disease onset [3,4].

Several studies have suggested that increased oxidative stress and inflammation are linked to the pathogenesis and progression of prion disease [5-8]. Investigations using animal models and cell culture have indirectly supported the involvement of oxidative stress in the pathogenesis of prion disease by showing that antioxidants can reduce neurotoxicity in cell cultures and animal models, as well as reports in which antioxidants have been shown to prevent the progression of prion disease [9,10]. Antibodies to N-methyl-D-aspartate receptors have been detected in the CSF of CJD patients, which are mostly seen in the context of autoimmune limbic encephalitis. In association with these findings suggesting neuroinflammatory involvement in sporadic CJD (sCJD), the pathogenic characteristics of cytokines has been investigated in prion-infected animal models [11]. Although it remains unclear whether there is a net increase in pro or anti-inflammatory cytokines in sCJD, a 2013 case-control study by Fujita et al. concluded that levels of pro-inflammatory cytokines interleukin (IL)-1 receptor antagonist and IL-17 were significantly elevated in the CSF of sCJD patients, thought to represent an early event in sCJD pathogenesis [12].

Of significance for the current case, *Ehrlichia chaffeensis* is a tick-borne rickettsial pathogen and the causative agent of human monocytic ehrlichiosis. Animal and canine studies have demonstrated that both wild-type Ehrlichia infection and vaccination with attenuated strains can induce significant IL-17 production, promoting a pro-inflammatory state [13]. In addition, platelet-rich plasma (PRP) injections can promote a local inflammatory response. Leukocyte-rich PRP formulations particularly have been demonstrated to activate tumor necrosis factor-alpha (TNF-a) and interferon-y with the subsequent activation of inflammatory signaling pathways [14,15]. The degree of inflammatory response is determined by the PRP preparation method and the baseline immunological and cytokine profile of the patient. Patients with pre-existing pro-inflammatory state, such as those with a chronic infection, may have PRP products with a greater concentration of inflammatory mediators [16].

To the best of our knowledge, there are no cases in the current literature of a correlational or triggering relationship among an Ehrlichiosis infection, PRP injections, and the development of sCJD. Given the rarity of this case, it is valuable to consider these elements of the patient history and their possible association with the development of sCJD. Recognition of chronic neuroinflammatory states among patients may prompt earlier consideration of sCJD as a potential differential diagnosis and initiation of appropriate counseling, infection control, and palliative support. Here, we offer the case of likely sCJD in an elderly male presenting with classic symptoms noted above, in the context of a chronic asymptomatic inflammatory state from tick-borne infection that was potentially exacerbated by a routine PRP injection.

## Case presentation

A Caucasian male in his 70s with relevant past medical history of hypertension, hyperlipidemia, cerebral vascular accident, and mild thrombocytopenia was transferred to our emergency department from an outside hospital for evaluation of acute encephalopathy. History was obtained by the patient’s spouse and adult children, who reported that the patient began demonstrating subtle cognitive decline days after a routine PRP injection for chronic hip pain three weeks ago. He became progressively confused with cessation of usual hobbies and loss of appetite accompanied by significant weight loss. Auditory or visual hallucinations were not reported. The patient notably spent significant time outdoors over the past several years golfing and lived adjoining woods. He did not have a history of cognitive impairment or psychiatric conditions. The family notably denied any recent travel, animal bites, or unusual dietary consumption. There was no reported family history of dementia or other neurologic conditions. His only medications included amlodipine, atorvastatin, and a multivitamin.

On physical exam, the patient was somnolent and mildly agitated. No rash was noted on full skin inspection. On neurological exam, he followed some commands intermittently with statement of name but was not oriented to time or place. No gaze deviation, ptosis, facial asymmetry, or facial sensory deficits were noted. He demonstrated significant left-sided weakness noted with prominent positive and negative myoclonus. He was diffusely hyperreflexic with positive crossed adductors and jaw reflexes.

**Figure 1:**
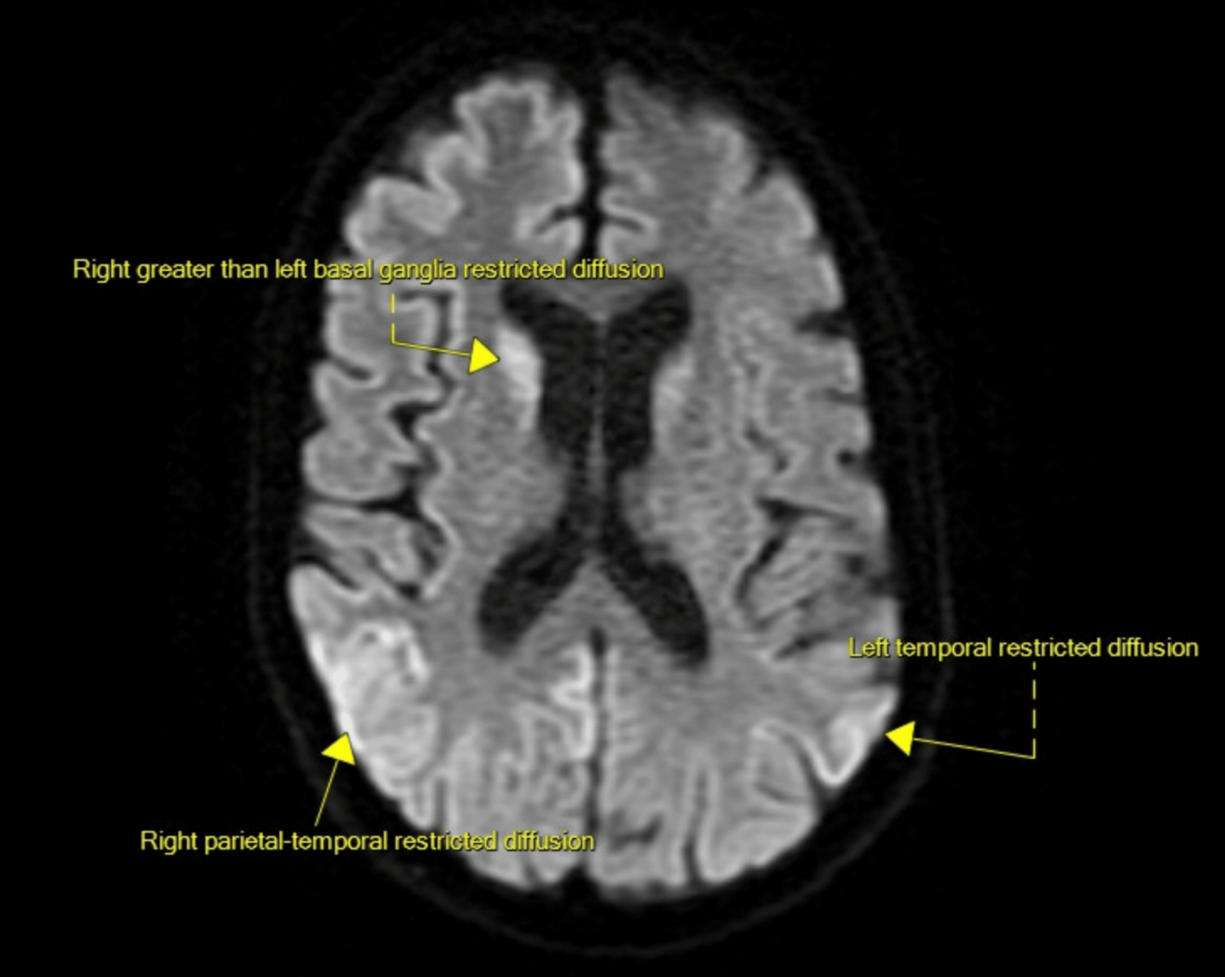
MRI brain with multifocal cortical diffusion signal abnormality and restricted diffusion of the right greater than left basal ganglia

Diffusion-weighted MRI showed multifocal cortical diffusion signal abnormality and restricted diffusion of the right greater than left basal ganglia, suggestive of encephalitis, postictal change, or CJD (Figure 1). CT abdomen pelvis did not show evidence of occult malignancy. Echocardiogram demonstrated preserved ejection fraction and no evidence of structural abnormalities or shunting. Daily complete blood counts were within normal limits apart from persistent mild thrombocytopenia with platelets ~120k dating back six years, at which time peripheral blood smear showed no abnormalities. There were no metabolic abnormalities on labs. He was started on IV acyclovir for presumed herpes simplex virus (HSV) encephalitis without appreciable improvement. He was observed on continuous EEG, which was notable for mixed theta/delta slowing but without changes suggestive of seizure activity.

He underwent lumbar puncture two days after admission. While awaiting lumbar puncture results, he received a five-day course of empiric IV immunoglobulin (IG) for possible autoimmune encephalitis without appreciable cognitive improvement. During his admission, his mentation continued to deteriorate with minimal verbal output, worsening diffuse positive and negative myoclonus, and diffuse hyperreflexia with increased tone. Enteral nutrition was initiated via nasogastric tube. 

CSF and infectious workup returned as: CSF culture no growth, venereal disease research laboratory (VDRL) CSF nonreactive, lyme disease negative, arbovirus CSF negative, West Nile virus Ab negative, HSV polymerase chain reaction (PCR) negative, varicella PCR negative, cryptococcal antigen CSF negative, serum protein electrophoresis within normal limits, syphilis antibody nonreactive, rabies antibody negative, heavy metal screen negative, dementia autoimmune/paraneoplastic negative. Incidentally, he was found to be Ehrlichia immunoglobulin G (IgG) positive with ferritin as 430 and mildly elevated erythrocyte sedimentation rate (ESR)/C-reactive protein (CRP). He was started on IV doxycycline for possible Ehrlichia encephalitis without appreciable improvement. The remaining CSF analysis later returned positive for prion disease, revealing positive real-time quaking-induced conversion (RT-QuIC) and elevated markers of neurodegenerative disease (T-tau protein and 14-3-3 gamma). sCJD was considered the primary driver of his rapidly progressive neurological decline. He died one week after discharge to hospice, within six weeks from disease onset.

## Discussion

The most likely diagnosis was sCJD given the subacute and rapidly progressive decline and neurological findings including myoclonus, hyperreflexia and MRI findings of multifocal cortical and basal ganglia diffusion restriction. The clinical course and imaging were suggestive of CJD, including the possible atypical subtype MV2K, which can also present with mixed cortical and deep grey matter involvement [4]. Viral encephalitis was considered in the setting of patient’s outdoor exposure and age, which can present with rapidly progressive encephalopathy and movement disorder. The absence of CSF pleocytosis and constitutional symptoms lowered the likelihood of this diagnosis. Autoimmune/paraneoplastic encephalitis were also considered. However, the lack of response to acyclovir or IVIG, as well as relative absence of psychiatric symptoms, made this diagnosis less likely. The diagnosis of anoxic brain injury on MRI was suggestive of a possible cerebrovascular event particularly given his history of recent transient ischemic attack (TIA). The subacute progression of symptoms, recent echocardiogram without structural abnormalities, and well-controlled hypertension and hyperlipidemia (low-density lipoprotein (LDL): 55) made hypoxic-ischemic encephalopathy unlikely.

Given the emerging association of neuroinflammatory states with the pathogenesis of sCJD, Ehrlichia IgG seropositivity is notable in this patient. As noted previously, neuroinflammation driven by upregulated inflammatory mediators and cytokines such as IL-17 and TNF-a is implicated in the pathogenesis and progression of sCJD. Rickettsial pathogens such as Ehrlichia are associated with inflammation, including significant IL-17 production by peripheral blood leukocytes. This patient’s frequent and prolonged time outdoors near woods, IgG seropositivity and elevated inflammatory biomarkers suggest a chronic Ehrlichia infection with a baseline asymptomatic neuroinflammatory state. His PRP products are, therefore, potentially more likely to have greater levels of inflammatory mediators, as noted in a recent 2023 study of PRP therapy by Niemann et al. [16]. We propose this patient’s repeated PRP injections may have sequentially stimulated pro-inflammatory pathways already stimulated by chronic Ehrlichia infection, increasing the risk of sCJD. The most recent injection likely precipitated his rapid cognitive decline.

sCJD is the most common form of prion disease, comprising 80-90% of cases characterized by rapid neurodegeneration, dementia and ataxia. Environmental, genetic and potentially anatomical factors are believed to be contributors; however, the exact pathogenesis remains largely unclear [17]. Prognosis is poor, with most patients surviving less than six months to one year after symptom onset [18]. Early recognition remains crucial to initiate timely infection control measures and to differentiate sCJD from other reversible causes of rapidly progressive dementia. 

Our case underscores several clinically relevant points of consideration. The patient’s rapidly progressive encephalopathy, in the setting of recent autologous PRP therapy and Ehrlichia IgG seropositivity, may enhance our understanding of several factors impacting sCJD pathogenesis linked by a unique patient presentation. As suggested by several studies, chronic neuroinflammation may underlie the development of sCJD. Pro-inflammatory cytokines such as IL-1 and IL-17 have been implicated in the CSF of sCJD patients, which is a relatively new finding in the study of prion disease [12]. The case-control study by Fujita et al. proposes that elevated pro-inflammatory cytokines may serve as an early event in the pathogenesis of sCJD rather than a consequence of it. As noted in a review by Burwinkel et al., inflammation and neurodegeneration can be driven by cytokines [11]. It is additionally notable that both vaccination and wild-type Ehrlichia infection can induce significant IL-17 production from activated CD4+ and gamma/delta T cells, giving rise to a pro-inflammatory state [13]. PRP injections, particularly leukocyte-rich formulations, have also been shown to promote activation of local inflammatory pathways [14,15]. Notably, the recent study by Dejnek et al. demonstrated autologous PRP formulated by one of the most commonly used PRP preparation systems can have a concentration of cytokines and other paracrine molecules above twice the baseline level in the patient’s serum [14]. Larger epidemiological studies are needed to determine if increase neuroinflammatory states are associated with CJD.

**Figure 2:**
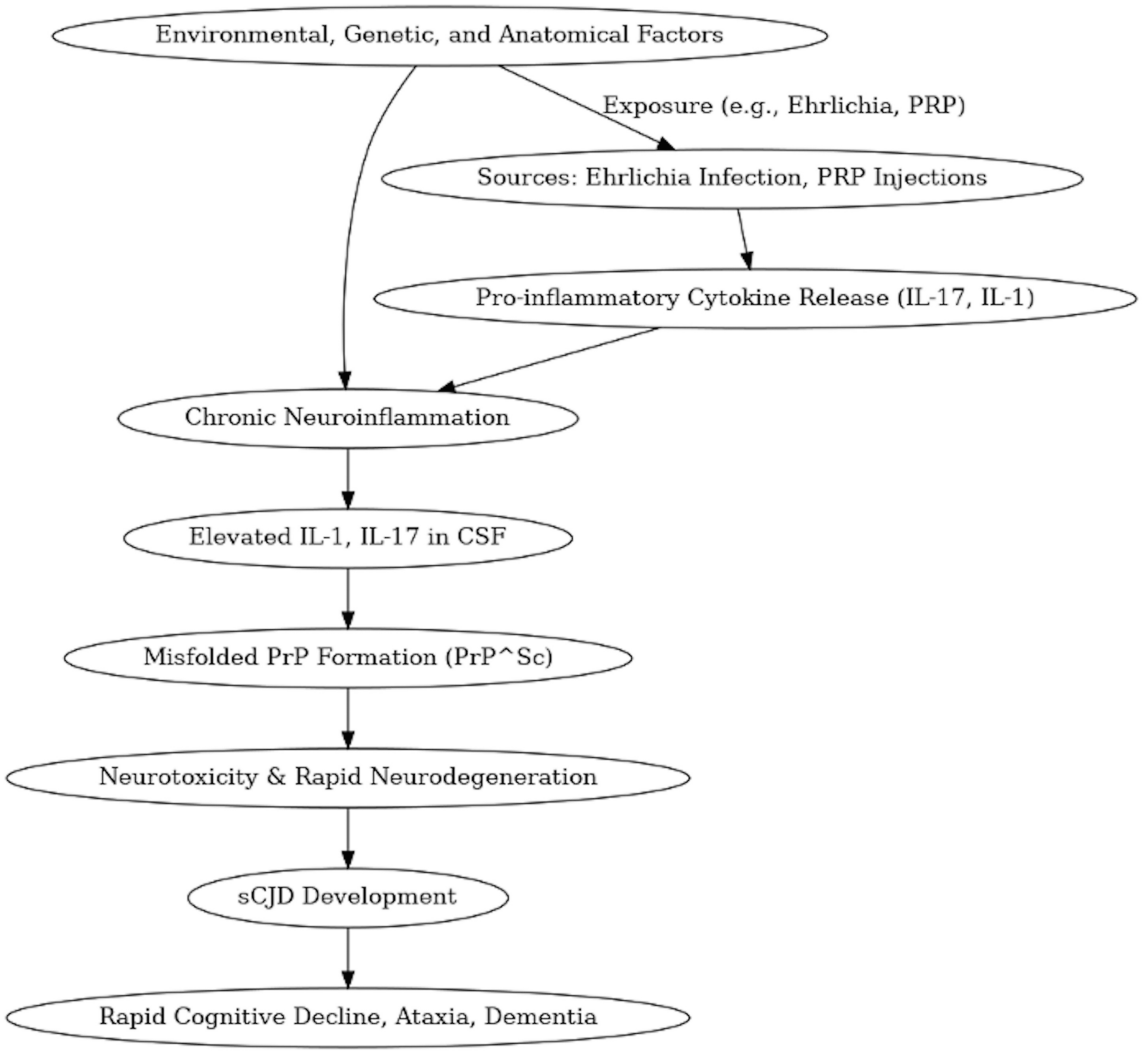
Proposed mechanism of sCJD pathogenesis PRP: Platelet-rich plasma; IL: Interleukin; PrP^Sc^: Misfolded prion protein; sCJD: Sporadic Creutzfeldt-Jakob disease

The current patient was an avid golfer and spent significant time outdoors, with high likelihood of repeat tick exposures. It is plausible he was sustaining a chronic asymptomatic neuroinflammatory state conducive to the development of sCJD in the setting of chronic Ehrlichia infection, further supported by the chronic thrombocytopenia noted on lab workup. This baseline inflammation was likely exacerbated by regular PRP injections, which were presumably rich in leukocytes and pro-inflammatory cytokines. The final injection, administered shortly before his cognitive decline, may have acted as at trigger-precipitating the rapid progression of sCJD. Our proposed mechanism for sCJD pathogenesis in this patient is summarized in Figure 2.

As noted above, sCJD pathogenesis is multifactorial, likely comprising of a decline in neuroprotective PRP, neurotoxicity of misfolded PrP and chronic neuroinflammation. While preventing the conversion of normal to misfolded PrP is a therapeutic goal, drugs that effectively block this conversion are not yet available for clinical use and immunotherapies remain under investigation [11]. Although targeting inflammatory responses would not provide a cure, it may prolong survival by curbing damaging secondary oxidative or inflammatory reactions. As suggested by Klein et al., interference with the IL-1 system may delay disease progression. Mice deficient in C1qa complement protein and mice deficient in the Fc receptor gamma chain were partially or fully protected against spongiform encephalopathy upon exposure to limiting amounts of prions. The later splenic accumulation of PrP^Sc^ was delayed, which suggested that disruption of normal inflammatory pathways may be protective against prion disease [19]. We, therefore, emphasize the importance of recognizing chronic neuroinflammatory states among patients as a possible risk factor for the development of sCJD and to consider it as a potential differential diagnosis in the case of rapid cognitive decline. 

## Conclusions

Chronic asymptomatic neuroinflammation may drive an eventual pathogenesis of sCJD. We emphasize that it is important to recognize potentially atypical factors that may contribute to an neuroinflammatory state, such as tick-borne infections or PRP injections. When sCJD is suspected, it may be beneficial to initiate anti-inflammatory therapeutic approaches in combination the standard counseling, infection control, and palliative support. In such cases the early initiation of anti-inflammatory therapeutic approaches, in combination with neuroprotective drugs, may enhance survival. 

## References

[1] Weber T (2000). Clinical and laboratory diagnosis of Creutzfeldt-Jakob disease. Clin Neuropathol.

[2] Kojima G, Tatsuno BK, Inaba M, Velligas S, Masaki K, Liow KK (2013). Creutzfeldt-Jakob disease: a case report and differential diagnoses. Hawaii J Med Public Health.

[3] Noor H, Baqai MH, Naveed H (2024). Creutzfeldt-Jakob disease: a comprehensive review of current understanding and research. J Neurol Sci.

[4] McGuire LI, Peden AH, Orrú CD (2012). Real time quaking-induced conversion analysis of cerebrospinal fluid in sporadic Creutzfeldt-Jakob disease. Ann Neurol.

[5] Brazier MW, Lewis V, Ciccotosto GD (2006). Correlative studies support lipid peroxidation is linked to PrPres propagation as an early primary pathogenic event in prion disease. Brain Res Bull.

[6] Crespo I, Roomp K, Jurkowski W, Kitano H, del Sol A (2012). Gene regulatory network analysis supports inflammation as a key neurodegeneration process in prion disease. BMC Syst Biol.

[7] Carroll JA, Striebel JF, Race B, Phillips K, Chesebro B (2015). Prion infection of mouse brain reveals multiple new upregulated genes involved in neuroinflammation or signal transduction. J Virol.

[8] Algarzae N, Hebron M, Miessau M, Moussa CE (2012). Parkin prevents cortical atrophy and Aβ-induced alterations of brain metabolism: ¹³C NMR and magnetic resonance imaging studies in AD models. Neuroscience.

[9] Asuni AA, Guridi M, Sanchez S, Sadowski MJ (2015). Antioxidant peroxiredoxin 6 protein rescues toxicity due to oxidative stress and cellular hypoxia in vitro, and attenuates prion-related pathology in vivo. Neurochem Int.

[10] Telling GC, Haga T, Torchia M, Tremblay P, DeArmond SJ, Prusiner SB (1996). Interactions between wild-type and mutant prion proteins modulate neurodegeneration in transgenic mice. Genes Dev.

[11] Burwinkel M, Riemer C, Schwarz A, Schultz J, Neidhold S, Bamme T, Baier M (2004). Role of cytokines and chemokines in prion infections of the central nervous system. Int J Dev Neurosci.

[12] Fujita K, Matsui N, Takahashi Y (2013). Increased interleukin-17 in the cerebrospinal fluid in sporadic Creutzfeldt-Jakob disease: a case-control study of rapidly progressive dementia. J Neuroinflammation.

[13] McGill JL, Nair AD, Cheng C, Rusk RA, Jaworski DC, Ganta RR (2016). Vaccination with an attenuated mutant of Ehrlichia chaffeensis induces pathogen-specific CD4+ T cell immunity and protection from tick-transmitted wild-type challenge in the canine host. PLoS One.

[14] Dejnek M, Moreira H, Płaczkowska S, Barg E, Reichert P, Królikowska A (2022). Leukocyte-rich platelet-rich plasma as an effective source of molecules that modulate local immune and inflammatory cell responses. Oxid Med Cell Longev.

[15] Anitua E, Zalduendo M, Troya M, Padilla S, Orive G (2015). Leukocyte inclusion within a platelet rich plasma-derived fibrin scaffold stimulates a more pro-inflammatory environment and alters fibrin properties. PLoS One.

[16] Niemann M, Ort M, Lauterbach L (2023). Individual immune cell and cytokine profiles determine platelet-rich plasma composition. Arthritis Res Ther.

[17] Shimamura MI, Satoh K (2025). Challenges and revisions in diagnostic criteria: advancing early detection of prion diseases. Int J Mol Sci.

[18] Connor A, Wang H, Appleby BS, Rhoads DD (2019). Clinical laboratory tests used to aid in diagnosis of human prion disease. J Clin Microbiol.

[19] Klein MA, Kaeser PS, Schwarz P (2001). Complement facilitates early prion pathogenesis. Nat Med.

